# Sex differences in outcomes of patients undergoing on-pump coronary artery bypass grafting surgery

**DOI:** 10.1371/journal.pone.0306902

**Published:** 2024-09-06

**Authors:** Débora Klein Ferreira, Aline Petracco Petzold, Rafael Braccio Zawislak, Jarbas Rodrigues de Oliveira, Mario Bernardes Wagner, Ricardo Medeiros Piantá, Renato Abdala Karam Kalil, Joao Carlos Vieira da Costa Guaragna, Luiz Carlos Bodanese

**Affiliations:** 1 Postgraduate Program in Medicine, Pontifícia Universidade Católica do Rio Grande do Sul, Porto Alegre, Rio Grande do Sul, Brazil; 2 School of Medicine, Pontifícia Universidade Católica do Rio Grande do Sul, Porto Alegre, Rio Grande do Sul, Brazil; 3 Department of Cardiovascular Surgery, Hospital São Lucas–Pontifícia Universidade Católica do Rio Grande do Sul, Porto Alegre, Rio Grande do Sul, Brazil; 4 Department of Surgery, Universidade Federal de Ciências da Saúde de Porto Alegre, Porto Alegre, Rio Grande do Sul, Brazil; 5 Department of Cardiology, Hospital Divina Providência, Porto Alegre, Rio Grande do Sul, Brazil; BSMMU: Bangabandhu Sheikh Mujib Medical University, BANGLADESH

## Abstract

There are controversies regarding the impact of sex on mortality and postoperative complications in patients undergoing on-pump coronary artery bypass grafting (CABG), although some studies demonstrate comparable outcomes. This study sought to evaluate sex differences regarding risk factors associated with hospital mortality and postoperative clinical outcomes among patients undergoing isolated on-pump CABG. We conducted a retrospective observational cohort study of patients who underwent isolated on-pump CABG from January 1996 to January 2020. Patients were divided into two groups (male and female) and compared regarding preoperative characteristics, surgical technical variables, and in-hospital outcomes. All-cause mortality between groups was compared using logistic regression. Risk factors for mortality, along with their respective odds ratios (OR), were separately assessed using a logistic regression model with p-values for interaction. We analyzed 4,882 patients, of whom 31.6% were female. Women exhibited a higher prevalence of age >75 years (12.2% vs 8.3%, p<0.001), obesity (22.6% vs 11.5%, p<0.001), diabetes (41.6% vs 32.2%, p<0.001), hypertension (85.2% vs 73.5%, p<0.001), and NYHA functional classes 3 and 4 (16.2% vs 11.2%, p<0.001) compared to men. Use of the mammary artery for revascularization was less frequent among women (73.8% vs 79.9%, p<0.001), who also received fewer saphenous vein grafts (2.17 vs 2.27, p = 0.002). A history of previous or recent myocardial infarction (MI) had an impact on women’s mortality, unlike in men (OR 1.61 vs 0.94, p = 0.014; OR 1.86 vs 0.99, p = 0.015, respectively). After adjusting for several risk factors, mortality was found to be comparable between men and women, with an OR of 1.20 (95% CI 0.94–1.53, p = 0.129). In conclusion, female patients undergoing isolated on-pump CABG presented with a higher number of comorbidities. Previous and recent MI were associated with higher mortality only in women. In this cohort analysis, female gender was not identified as an independent risk factor for outcome after CABG.

## Introduction

Coronary artery disease is the leading cause of mortality worldwide, and coronary artery bypass grafting (CABG) surgery is a well-established and widely accepted treatment option, especially in advanced coronary artery disease [1]. A growing body of research has been dedicated to exploring the factors that could impact the outcomes of CABG, revealing some sex-based disparities in patient profiles and clinical outcomes.

Controversy persists regarding the influence of sex in patients undergoing isolated CABG, particularly concerning risk factors associated with mortality, in-hospital outcomes, and postoperative complications, despite some studies reporting comparable mortality rates. Some authors found female sex to be associated with higher operative mortality in CABG, even after adjusting for risk factors, suggesting that women may represent an independent risk factor for worse outcomes in this context [2–10]. Other observational studies have reported divergent results, showing similar mortality and outcomes between sexes when adjusted for different factors [11–26]. This has led to the hypothesis that female sex may involve particularities that influence outcomes, not necessarily that sex itself represents a risk factor for worse outcomes.

In this context, the objective of the present study is to analyze sex differences in patients undergoing isolated CABG, assessing clinical characteristics, intraoperative aspects, postoperative complications, and in-hospital mortality. Additionally, the study aims to conduct a comprehensive analysis of potential risk factors associated with in-hospital mortality after CABG, focusing on understanding differences between sexes.

## Methods

This is a retrospective observational cohort analysis of consecutive adult patients who underwent on-pump CABG from January 1996 to January 2020 at a tertiary hospital in Brazil. The patients’ medical records were retrospectively reviewed, and the data were accessed on multiple days during data collection from January to June 2022 for research purposes. All data were anonymized before the review so that the authors had no access to information that could identify individual participants during or after data collection. Only isolated CABG was considered; those who underwent valve repair, combined valve and CABG surgery, or congenital heart defect repairs were excluded. All procedures were performed under extracorporeal circulation, with cardioplegia achieved using hypothermic blood St. Thomas’ Hospital solution. After surgery, all patients were transferred to the Postoperative Cardiac Surgery Intensive Care Unit and placed on mechanical ventilation. Patients were followed throughout their hospital stay by the Cardiac Surgery postoperative management team, and their progress recorded in a database until hospital discharge.

The patients were divided into two groups (male and female) and the profiles of each group analyzed regarding preoperative characteristics: age, angina class, New York Heart Association (NYHA) functional class, surgery type (elective, urgent, or emergent), history of atrial fibrillation (AF), history of stroke, diabetes, peripheral arterial disease (PAD), history of cardiac surgery, smoking, history of percutaneous coronary intervention (PCI), chronic obstructive pulmonary disease (COPD), hypertension, dyslipidemia, obesity (defined as body mass index [BMI] ≥ 30 kg/m^2^), ejection fraction (EF) (measured by echocardiography or scintigraphy), creatinine, chronic kidney disease (CKD), hemodialysis, use of preoperative intra-aortic balloon pump (IABP), and preoperative medication use, including calcium channel blockers, antiplatelet agents, antiarrhythmic agents, beta-blockers, digoxin, diuretics, statins, angiotensin-converting enzyme (ACE) inhibitors, insulin, and nitrates. Recent myocardial infarction (MI) was defined as acute MI that prompted hospitalization or occurred less than 30 days before hospitalization; previous MI was defined as acute MI occurring more than 30 days before the hospitalization that prompted CABG surgery. Unstable angina was defined as clinical presentation of acute coronary syndrome in the absence of positive biomarkers of myocardial necrosis or ST segment elevation on electrocardiogram. The extent of coronary artery disease (CAD) was classified by territory: left main coronary artery, anterior descending artery, circumflex artery (including marginal branch), diagonal artery (including diagonal branch), and right coronary artery (including posterior descending artery and posterolateral branch). We utilized the internationally recognized risk score, EuroSCORE II, and a locally developed risk score, the Guaragna score, to compare groups [27].

The surgical technical variables of interest were duration of cardiopulmonary bypass (CPB), aortic clamping time, the use of mammary artery and the number of saphenous vein grafts. We also evaluate the coronary arteries revascularized with saphenous vein divided by territory, including anterior descending, circumflex (including marginal branch), diagonal (including diagonal branch), right (including posterior descending artery and posterolateral branch). Incomplete revascularization was defined by the surgeon upon ICU arrival, generally when there was a lesion in a major coronary artery that could not be revascularized.

The primary outcome was in-hospital mortality, defined as death from any cause during the hospitalization in which the surgical procedure was performed. The other outcomes evaluated were postoperative MI (defined as an increase in at least 1 of 2 measurements of cardiac markers above the 99th percentile for the population plus typical chest pain, ECG changes, or evidence of necrosis on cardiac imaging), atrial fibrillation, complete atrioventricular block, stroke (defined as any new neurological deficit persisting for more than 24 hours, confirmed by clinical examination by a neurologist and brain imaging—computed tomography or magnetic resonance imaging), a composite of clinical pulmonary complications (pneumonia, atelectasis, acute respiratory failure, prolonged mechanical ventilation), renal complications (acute renal failure, acute-on-chronic renal failure, and/or need for hemodialysis in a patient who was previously not dialytic), increased bleeding (more than 2 mL/kg per hour in the first 3 to 12 hours after surgery), reintervention (for bleeding usually in the presence of hemodynamic instability and/or when bleeding is greater than 500 mL in the first hour, or greater than 400 mL per hour in 3 hours, or greater than 300 mL per hour in 3 hours), mediastinitis, length of ICU stay, length of hospital stay, and combined major cardiovascular events (MACE) (all cause death, MI, and stroke). Some missing and conflicting data for primary variables were backfilled and validated via record linkage to the patient hospital record. Valid analyzed data were displayed on the same line as the variable. Missing continuous variables data needed for risk scores calculation were imputed with the median.

Continuous variables were described as means ± standard deviations and compared using Student’s *t*-test. Categorical variables were described as absolute and relative frequencies and compared using the chi-square test. Parametric data were compared using Student’s t-test for up to two variables and Fisher’s exact test for more than two variables. Nonparametric data were evaluated using the Mann–Whitney *U* test.

The initial consideration of variables followed a hierarchical model based on biological plausibility and information from the existing literature regarding the relevance and strength of associations between potential risk factors and mortality. Once these variables were listed, a multiple logistic regression was performed using a forward stepwise process. Mortality between groups was compared using logistic regression. Risk factors for mortality between groups were compared using a binary logistic regression model, in which unadjusted odds ratios (OR) were calculated for each risk factor separately between the two groups and compared using p-values for interaction. The data were processed and analyzed in SPSS Version 22.0 and R Studio.

This research project was guided by the principles set forth in the Declaration of Helsinki and was approved by the Research Ethics Committee of the PUCRS School of Medicine (registration number 3953223). Since this was a retrospective study, the requirement for informed consent was waived.

## Results

From 7,166 consecutive patients undergoing Cardiac Surgery, 5,302 underwent any coronary revascularization, and 4,882 patients underwent isolated CABG. The study analyzed 4,882 patients who underwent isolated CABG, of whom 1,547 (31.6%) were female. Women had a higher mean age (>75 years: 12.2% vs 8.3%, p<0.001) and a higher prevalence of obesity (22.6% vs 11.5%, p<0.001), diabetes (41.6% vs 32.2%, p<0.001), and hypertension (85.2% vs 73.5%, p<0.001). Women also had a higher prevalence of worse NYHA functional class (class 3 and 4: 16.2% vs 11.2%, p<0.001), a higher incidence of unstable angina (42.2% vs 35.3%, p<0.001), and higher risk scores (EuroSCORE II: 2.26 vs 1.62, p<0.001; Guaragna score: 6.15 vs 4.06, p<0.001). Men presented with a higher prevalence of smoking (34.3% vs 27%, p<0.001), COPD (16.5% vs 11.6%, p<0.001), PAD (11.8% vs 8.7%, p = 0.001), and renal failure (16.0% vs 9.4%, p<0.001), as well as a lower mean ejection fraction (53.4% vs 55.3%, p<0.001) (Table 1).

**Table 1:** Preoperative clinical characteristics stratified by sex.

Variables	Female	Male	*p*
	n = 1,547	n = 3,335	
**Age (years)**			
**Mean ± SD**	62.3 ± 10.2	61.5 ± 9.5	0.004
**<40**	20 (1.3)	37 (1.1)	<0.001
**40–60**	559 (36.1)	1,324 (39.7)	
**60–75**	780 (50.4)	1,694 (50.8)	
**≥75**	188 (12.2)	278 (8.3)	
**Diabetes**	644 (41.6)	1,074 (32.2)	<0.001
**Hypertension**	1,381 (85.2)	2,451 (73.5)	<0.001
**Creatinine (mean ± SD)**	1.06 ± 0.52	1.25 ± 0.67	<0.001
**Atrial fibrillation**	44 (2.8)	102 (3.1)	0.719
**PAD**	134 (8.7)	394 (11.8)	0.001
**Stroke**	108 (7.0)	250 (7.5)	0.560
**Dyslipidemia**	456 (29.5)	892 (26.7)	0.051
**COPD**	179 (11.6)	550 (16.5)	<0.001
**Smoking**	417 (27.0)	1,144 (34.3)	<0.001
**Obesity**	349 (22.6)	382 (11.5)	<0.001
**CKD**	146 (9.4)	534 (16)	<0.001
**Hemodialysis**	18 (1.2)	51 (1.5)	0.363
**Recent MI**	295 (19.1)	609 (18.3)	0.501
**Previous MI**	720 (46.5)	1,644 (49.3)	0.074
**HF NYHA**	**n = 1,513**	**n = 3,268**	
**Class I**	945 (62.5)	2,272 (69.5)	<0.001
**Class II**	323 (21.3)	629 (19.2)	
**Class III**	186 (12.3)	291 (8.9)	
**Class IV**	59 (3.9)	76 (2.3)	
**Angina—n**	**n = 1,537**	**n = 3,299**	<0.001
**Asymptomatic**	264 (17.2)	697 (21.1)	
**Class 1**	65 (4.2)	204 (6.2)	
**Class 2**	235 (15.3)	634 (19.2)	
**Class 3**	305 (19.8)	570 (17.3)	
**Class 4**	20 (1.3)	30 (0.9)	
**Unstable**	648 (42.2)	1,164 (35.3)	
**Previous PCI**	247 (16.0)	547 (16.4)	0.739
**Previous cardiac surgery**	27 (1.7)	84 (2.5)	0.099
**Nature of surgery**	**n = 1546**	**n = 3335**	0.003
**Elective**	1437 (92.9)	3178 (95.3)	
**Urgent**	95 (6.1)	133 (4.0)	
**Emergency**	14 (0.9)	24 (0.7)	
**Previous medication use**			
**Calcium channel blocker**	308 (19.9)	583 (17.5)	0.042
**Antiplatelet**	715 (46.2)	1,558 (46.7)	0.769
**Antiarrhythmic**	31 (2)	76 (2.3)	0.613
**Beta-blocker**	1,126 (72.8)	2,371 (71.1)	0.236
**Digoxin**	89 (5.8)	147 (4.4)	0.049
**Diuretic**	414 (26.8)	653 (19.6)	<0.001
**Statin**	1.011 (65.4)	2,299 (68.9)	0.014
**ACE inhibitor**	726 (46.9)	1,483 (44.5)	0.115
**Insulin**	157 (10.1)	229 (6.9)	<0.001
**Nitrate**	929 (60.1)	1,787 (53.6)	<0.001
**Preoperative IAB**	141 (9.1)	354 (10,6)	0.118
**Ejection fraction**	**n = 1546**	**n = 3335**	
**Mean ± SD**	55.3 ± 13.9	53.4 ± 13.8	<0.001
**<40%**	214 (13.8)	551 (16.5)	0.018
**≥40%**	1,333 (86.2)	2,784 (83.5)	
**Extent of CAD**	**n = 1546**	**n = 3335**	
**Left main**	416 (26.9)	998 (29.9)	0.030
**LAD**	1,387 (89.7)	2,996 (89.8)	0.849
**Circumflex**	115 (72.1)	2,553 (76.6)	0.001
**Diagonal**	498 (32.2)	1,216 (36.5)	0.004
**Right**	1,158 (74.9)	2,589 (77.6)	0.033
**Number of diseased coronary arteries—n (%)**	**n = 1546**	**n = 3335**	<0.001
**1**	105 (6.8)	189 (5.7)	
**2**	306 (19.8)	545 (16.3)	
**3**	615 (39.8)	1,342 (40.2)	
**≥4**	445 (28.8)	1,130 (33.9)	
**EuroSCORE II—n**	**n = 1546**	**n = 3335**	<0.001
**Mean ± SD**	2.26 ± 2.05	1.62 ± 1.64	
**Guaragna score—n**	**n = 1546**	**n = 3335**	<0.001
**Mean ± SD**	6.15 ± 5.02	4.06 ± 4.67	

Categorical variables are presented as n (%), continuous variables as mean ± standard deviation, and skewed variables as mean and percentiles.

SD = standard deviation; PAD = peripheral artery disease; COPD = chronic obstructive pulmonary disease; CKD = chronic kidney disease; MI = myocardial infarction, HF NYHA = New York Heart Association functional classification for heart failure; PCI = percutaneous coronary intervention; ACE = angiotensin-converting enzyme; IAB = intra-aortic balloon; LAD = left anterior descending artery.

Throughout the entire cohort, the mean utilization of the mammary artery for revascularization was less common in women (73.8% vs. 79.7%, p<0.001), as shown in Table 2. On the other hand, use of the saphenous vein for revascularization of the anterior descending artery was more common in females (22.9% vs. 16.9%, p<0.001). Women also received fewer saphenous grafts (2.17 vs. 2.27, p = 0.002), corresponding to revascularization of the territory of the circumflex artery (71.4% vs. 74%, p = 0.050), diagonal artery (41.7% vs. 46.9%, p = 0.001), and right coronary artery (64.8% vs. 67.9%, p = 0.031). Additionally, the mean duration of CPB and the mean aortic cross-clamp time were shorter in women (82.6 vs. 88.0 min, p<0.001 and 49.6 vs. 53.1 min, p<0.001, respectively).

**Table 2:** Operative characteristics stratified by sex.

Variable	Female	Male	*p*
	n = 1,547	n = 3,335	
**Incomplete revascularization**	134 (8.7)	274 (8.2)	0.617
**Use of mammary artery**	1,141 (73.8)	2,665 (79.9)	<0.001
**Saphenous vein graft**	**n = 1511**	**n = 3267**	
**LAD**	354 (22.9)	565 (16.9)	<0.001
**Circumflex**	1,105 (71.4)	2,471 (74.1)	0.050
**Diagonal**	645 (41.7)	1,563 (46.9)	0.001
**Right**	1,002 (64.8)	2,264 (67.9)	0.031
**Number of saphenous vein grafts (Mean ± SD)**	2.17 ± 0.92	2.27 ± 0.89	0.002
**CBP time**	82.6 ± 29.1	88.0 ± 32.2	<0.001
**Aortic clamping time**	49.6 ± 22.6	53.1 ± 24.7	<0.001

Categorical variables presented as n (%), continuous variables as mean ± standard deviation.

LAD = left anterior descending artery; CPB = cardiopulmonary bypass.

Postoperative complications that were more prevalent in women included complete atrioventricular block (9.7% vs. 7.6%, p = 0.014) and MACE (23.3% vs. 19.1%, p = 0.001). Conversely, men had a higher incidence of increased bleeding (22.2% vs. 16.5%, p<0.001). Women had a longer mean hospital stay (20.79 vs. 20.19 days, p<0.001) (Table 3).

**Table 3:** In-hospital clinical outcomes stratified by sex.

Variable	Female	Male	*p*
	n = 1,547	n = 3,335	
**Myocardial infarction**	225 (14.5)	422 (12.7)	0.077
**Atrial fibrillation**	292 (18.9)	679 (20.4)	0.232
**Complete AV block**	150 (9.7)	252 (7.6)	0.014
**Stroke**	56 (3.6)	101 (3.0)	0.295
**MACE**	360 (23.3)	638 (19.1)	0.001
**Pulmonary**	673 (43.5)	1.372 (41.1)	0.119
**Renal**	194 (12.5)	412 (12.4)	0.852
**Increased bleeding**	255 (16.5)	739 (22.2)	<0.001
**Reintervention**	54 (3.5)	152 (4.6)	0.092
**Mediastinitis**	38 (2.5)	591 (2.7)	0.632
**Length of stay in ICU (days)—n**	**795**	**1797**	
**Mean ± SD**	4.63 ± 7.68	4.40 ± 7.2	0.148
**Postoperative length of stay—n**	**1542**	**3327**	0.007
**Mean ± SD**	10.75 ± 10.12	10.87 ± 12.72	
**Length of total hospitalization (days)—n**			
**Mean ± SD**	20.76 ± 12.95	20.16 ± 15.08	<0.001
**Death**	159 (10.3)	231 (6.9)	<0.001

Categorical variables presented as n (%), continuous variables as mean ± standard deviation, and asymmetric variables as mean and percentiles.

AV = atrioventricular; MACE = major cardiovascular events (death, myocardial infarction, and stroke); ICU = Intensive care unit.

In our cohort, age, NYHA class 3 and 4, and urgent/emergency surgery were all factors associated with increased mortality in both sexes. Additionally, the use of mammary artery grafts for revascularization was associated with lower mortality (OR 0.59, 95% CI 0.46–0.76; p<0.001) (Table 4). Following adjustment for various factors, no significant difference in mortality was observed between men and women, with an odds ratio (OR) of 1.20 (95% CI 0.94–1.53; p = 0.129).

**Table 4:** Adjusted multivariate predictors for in-hospital mortality.

Variable ^a^	OR _a_	95%CI	*p*
**Female**	1.20	0.94–1.53	0.129
**Age ≥ 65 years**	2.32	1.83–2.94	<0.001
**Angina class 4 and unstable**	0.94	0.73–1.21	0.636
**NYHA class 3–4**	2.39	1.84–3.11	<0.001
**Diabetes**	1.18	0.93–1.50	0.182
**Obesity**	1.27	0.93–1.73	0.132
**Hypertension**	1.05	0.78–1.39	0.757
**Previous MI**	0.99	0.76–1.29	0.967
**Recent MI**	1.11	0.80–1.55	0.522
**Urgent/emergent**	12.31	8.98–16.87	<0.001
**Use of mammary artery**	0.59	0.46–0.76	<0.001
**Incomplete revascularization**	0.75	0.48–1.15	0.186

^**a**^ Missing 61 cases;

**OR**_**a**_ = adjusted odds ratio; CI = confidence interval; NYHA = New York Heart Association; MI = myocardial infarction.

**Fig 1:**
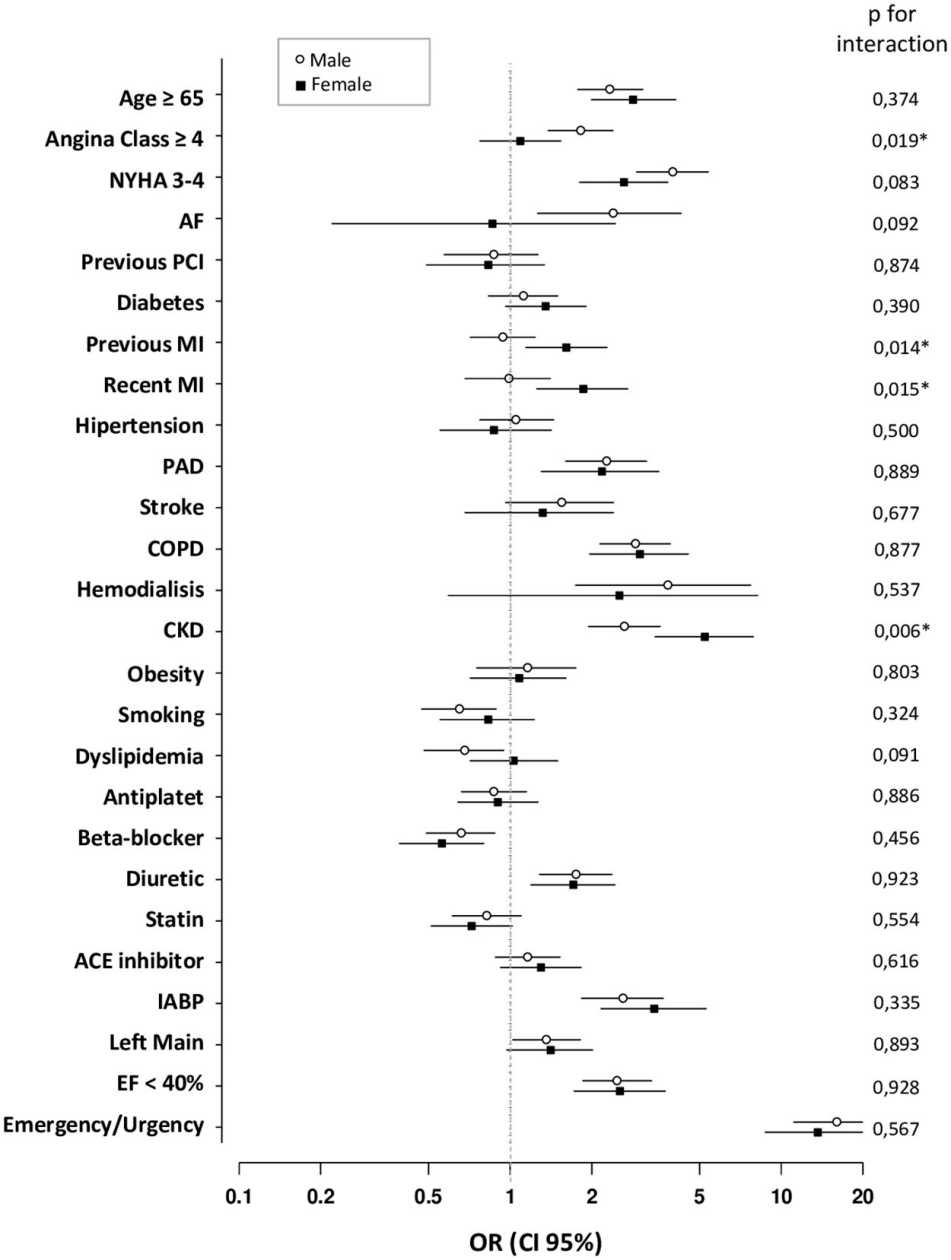
Sex-specific odds ratio for mortality risk. NYHA = New York Heart Association functional class; PCI = percutaneous coronary angioplasty; MI = myocardial infarction; PAD = peripheral arterial disease; COPD = chronic obstructive pulmonary disease; CKD = chronic kidney disease; ACE = angiotensin-converting enzyme; IABP = intra-aortic balloon pump; LMCA = left main coronary artery; EF = ejection fraction; OR = odd ratio; 95% CI = 95% confidence interval.

In our analysis of risk factors associated with mortality in each group separately (Fig 1), we observed that previous MI had a significant impact on mortality in women (OR 1.61 vs 0.94; p = 0.014), and the same trend was noted for recent MI (1.86 vs 0.99; p = 0.015). Conversely, class 4 angina or unstable angina was a factor influencing mortality more in men than in women (1.82 vs 1.09; p = 0.019).

**Fig 2:**
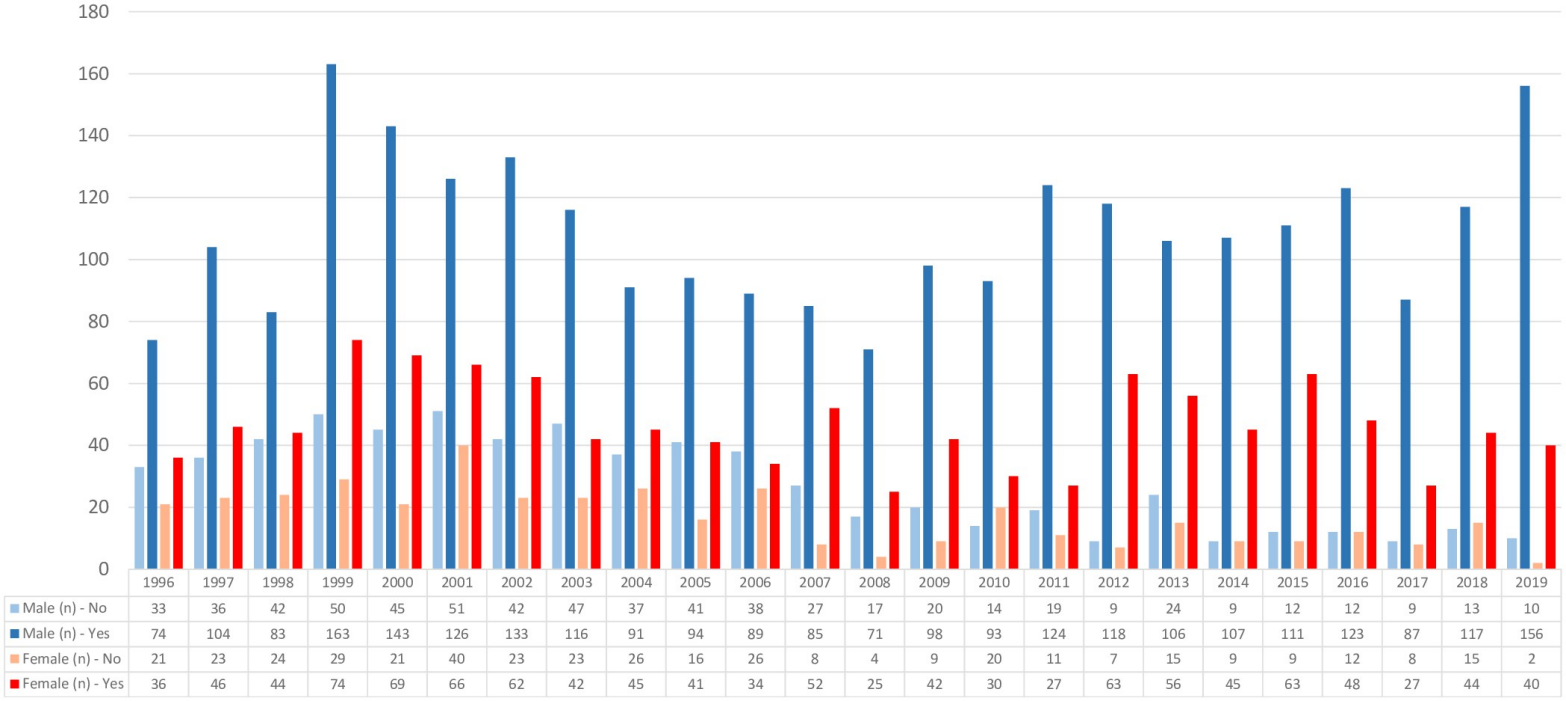
Internal mammary artery utilization rate by sex per year.

Additionally, we analyzed the time trend of mammary artery usage, comparing it by sex (Fig 2). It revealed that mammary artery usage remained higher in males, but its occurrence increased over the years: from 69.1% vs 63.1% in 1996 to 93.9% vs 95.2% in 2019 for males and females, respectively.

## Discussion

In the present study, females were older on average than males, with a greater proportion of women above 75 years of age. We also found an association of age >65 years with mortality (OR 2.42; p<0.001) for both sexes. In most previous studies comparing outcomes between sexes, higher age was found in women, which was associated with increased mortality [2, 3, 12, 14, 28–31]. One of the reasons for this finding is that women tend to manifest coronary artery disease later than men, and consequently seek surgical treatment approximately 10 to 20 years later [19]. The data of the present study also indicate that women presented with more advanced ischemic disease, as evidenced by worse NYHA functional class and angina, higher use of nitrates, diuretics, and digoxin, and a higher proportion of urgent and emergency surgery. These results are consistent with previous studies [21, 28, 32–34] and contributes to the theory that women are referred for surgery later.

The hormonal influence of estrogen in women may exert a protective effect against atherosclerosis by improving endothelial function and antioxidative activity, ultimately preventing the formation and thrombosis of atherosclerotic plaques [3], potentially delaying the onset of cardiovascular disease compared to men. Moreover, women may harbor greater apprehension towards the disease and the procedures involved in its treatment, potentially leading to delayed referral for both diagnosis and treatment [3].

In women, we found a higher prevalence of hypertension, obesity, diabetes mellitus, and insulin use, probably associated with the metabolic syndrome. The presence of diabetes mellitus has a particular impact on women, potentially eliminating the hormonal protective factor and increasing the odds of developing CAD, leading to a worse outcome [2, 3, 6, 12, 14, 28–30, 35]. The hormonal changes associated with pregnancy, ovarian cycles, post menopause, and the polycystic ovary syndrome can lead to the emergence of risk factors of cardiovascular risk factors such as hypertension, abdominal obesity, dyslipidemia, and insulin resistance. These comorbidities may have a greater impact on cardiovascular health than menopause itself [32]. Consequently, in addition to CAD often manifesting later in women, it frequently presents with a higher prevalence of cardiovascular risk factors when associated with metabolic syndrome.

In terms of surgical strategy, we observed a lower mean utilization of the mammary artery as a graft in women, consistent with findings from previous studies [3, 13, 15, 18, 21, 28, 30, 36–38]. Additionally, we found a higher usage of saphenous vein graft to LAD in women. One possible explanation for these differences is that women may have smaller coronary artery diameters [2, 39], which has been previously associated with higher in-hospital mortality rates in patients undergoing CABG. Furthermore, smaller coronaries may be related to a lower number of anastomoses due to technical difficulties during surgery, as well as a higher risk of graft thrombosis [36]. Moreover, in women, larger breast size may exert tension on the skin and surgical wound in the sternum, potentially leading to decreased circulation and increased risk of mediastinitis [40]. Local factors, combined with smaller vessels and a possibly more fragile sternum, may predispose women to sternal dehiscence, which could explain the lower use of mammary artery grafts in women.

In this study, women had a lower mean frequency of IMA and vein grafts utilization in compared to men, despite similar rates of incomplete revascularization among both groups. This trend implies that women may have required fewer grafts overall, which could signify also less severe epicardial coronary disease in this population. Mickleborough et al. [15] support this theory with their observation of a greater difficulty in visualizing the coronary arteries in women, attributed to smaller diameters and more distal extension of coronary artery disease. They also reported higher mortality in patients with distal coronary artery disease, irrespective of sex. This microvascular involvement may also explain more advanced symptoms in women, despite less epicardial coronary disease and preserved EF, even when presenting with more advanced ischemic disease [11]. We also observed that use of the mammary artery increased in both sexes over time. This can be explained by the 25-year study period: at the beginning of the cohort, IMA grafts were not used as routinely as they are currently.

We observed that the use of mammary artery grafts was a protective factor for mortality in both sexes. Thus, the less usage in women may represent an important factor impacting on outcomes. Therefore, it seems that women do not receive the same treatment as men, despite guidelines recommending the use of mammary artery grafts for surgical revascularization in both sexes. One explanation for this difference could be operator bias, whereby the surgeon may anticipate that female patients have a higher risk of poor outcomes and therefore do not offer the best option (mammary artery grafts), especially in cases with narrower coronaries [32, 37].

When evaluating factors that independently influence mortality by sex, we found that previous and recent MI had a significant impact on mortality specifically in women. Although no previous studies employing the same analysis were found, certain hypotheses can be proposed regarding differences in the behavior of coronary artery disease between sexes. CAD in women may be more silent, with atypical clinical presentations, making diagnosis may be more challenging and delayed [32, 41, 42]. Thus, it is plausible that the disease is not being diagnosed early enough. Ram et al. observed distinct clinical and anatomical presentations in women with acute coronary syndrome: women more frequently present with eccentric plaques, endothelial dysfunction, more plaque erosion and thrombosis compared to men, who more frequently present with plaque rupture as the primary mechanism of acute coronary syndrome [33]. It may also be suggested that a history of myocardial infarction before CABG could indicate a poorer prognosis for women, as patients with a prior infarction may be in worse clinical condition for surgery. Additionally, some authors have suggested that female patients receive less secondary prevention after a myocardial infarction event, potentially contributing to worse outcomes in those with a history of previous MI [29, 33, 43]. Consequently, when surgery is indicated in a patient with a previous MI, it likely signifies more advanced disease, further exacerbated by delayed diagnosis.

In our study, women exhibited a higher incidence of complete AV block, MACE and an extended duration of hospitalization, consistent with findings from other studies [44]. Sattartabar et al. identified several risk factors associated with MACE, including diabetes, hypertension, and dyslipidemia, in both sexes [5]. The higher incidence of complications in females may be attributed to a greater number of risk factors in women.

Similar mortality rates were observed in women compared to men after adjusting for different factors. Prior studies have reported conflicting results, with some identifying female sex as an independent risk factor for mortality even after adjustments [3–5, 14, 28–30, 32–34, 36, 41, 45, 46], while others report comparable mortality between men and women after adjusting for variables [11–15, 17–21, 23, 47, 48]. In a meta-analysis published by Robinson et al. [45], which compared observational studies, 13 studies identified female sex as an independent factor for mortality, while 8 others found no difference after the same adjustments. The included studies exhibited relatively high heterogeneity, making it challenging to generalize the findings. El-Andari et al., in a review article, concluded that women presented with increased comorbidities and risk factors, and this was probably accounting for the worse outcomes [49]. Furthermore, it was previously found that high-performance hospitals had lower differences in mortality between sexes when compared to those with lower surgical volume [41]. Accordingly, hospitals and teams that integrate and visualize differences in comorbidities in women, with a more detailed surgical and perioperative plan, can reduce sex disparities in outcomes even in hospitals with a lower procedure volume.

In the present cohort, mortality rates were found compared to recent studies in the Brazilian population, which reported rates ranging from 2.6% to 13.1% across Brazilian states over a 10-year period) [50]. Additionally, the majority of patients undergoing CABG, despite their procedures being considered elective, entered the hospital through the emergency department, primarily due to challenges in accessing the public healthcare system. Patients were kept in hospital while awaiting scheduling of the procedure, even when admitted through the emergency department or referred from smaller outside institutions, as evidenced by the relatively extended mean length of stay. It should also be noted that, in this study, hospital mortality encompassed late mortality if it occurred during the same hospitalization. It is crucial to emphasize that variations based on region, risk factors, and access to healthcare can lead to differences approaches and treatment outcomes, both in the general population and, specifically, in female patients in this country.

This study has limitations. Firstly, it is retrospective, which inherently limits the collection and analysis of data after the outcomes were recorded. Additionally, its single-center design and moderately sized sample only represent the local population of southern Brazil and may not be generalizable to the country as a whole due to heterogeneity of the different regions, or to other populations and territories. Furthermore, our risk variables and outcomes may differ from other datasets such as the Society of Thoracic Surgeons, making it difficult to generalize findings.

On the other hand, this study has certain advantages, including more consistent clinical and surgical practice and rigorous data collection spanning 25 years under the same medical coordinators. The sample reflects the real-life scenario of a university-affiliated tertiary referral center with a medical residency program, potentially serving as a representative model for other hospitals in underdeveloped countries facing similar conditions. Finally, the sampling of patients from a specific region contributes to a better understanding of the local population and can facilitate improvement of local treatment strategies.

Future research could benefit from larger, more diverse samples and longitudinal designs to further explore the relationships identified in this study. Given the differences among populations, it is also important to encourage the collection of databases to explore differences between groups. This can aid in developing risk scores to assist in selecting the optimal treatment options for each patient.

We understand that the findings of the present study serve as a warning for cardiovascular disease in women, who often present later for treatment, possibly due to their more silent clinical behavior, difficulty accessing the healthcare system, and lack of information about the condition. It becomes imperative to pay greater attention to this patient group, considering the importance of cardiovascular disease, associated risk factors, and its complications, including mortality, aiming to reduce relevant clinical outcomes and improve quality of life. Therefore, it is necessary to improve primary and secondary prevention strategies and the treatment of coronary artery disease, so that patients can be treated more appropriately and at an earlier stage.

## Conclusion

In our analysis, women exhibited a higher comorbidity burden. The most prevalent complications in women included complete AV block and MACE. Notably, a history of remote or recent myocardial infarction had a more pronounced impact on surgical prognosis exclusively in women. Following adjustment for risk factors, mortality rates were comparable between males and females.

Our findings offer further evidence of an elevated risk profile in women undergoing on-pump CABG. Moreover, these results support the hypothesis that the characteristics of female candidates for CABG, rather than sex itself, may account for poorer outcomes.

## Supporting information

S1 File Guarana score calculation. (DOCX)

S2 File P-value for interaction of factors associated with mortality. (DOCX)
