# Supplementary material for: Sex differences in outcomes of patients undergoing on-pump coronary artery bypass grafting surgery
Source: PLoS One. 2024 Sep 6;19(9):e0306902. doi: 10.1371/journal.pone.0306902 (PMC11379269; doi:10.1371/journal.pone.0306902)
Supplement: S1 File — (DOCX) [file pone.0306902.s001.docx]

Guaragna Risk Score


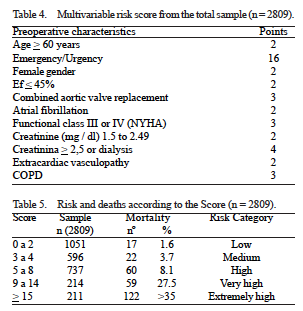


Cadore MP, Guaragna JC, Anacker JF, Albuquerque LC, Bodanese LC, Piccoli J da C, et al. A score proposal to evaluate surgical risk in patients submitted to myocardial revascularization surgery. Rev Bras Cir Cardiovasc. 2010;25:447-456
