# Supplementary material for: Sex differences in outcomes of patients undergoing on-pump coronary artery bypass grafting surgery
Source: PLoS One. 2024 Sep 6;19(9):e0306902. doi: 10.1371/journal.pone.0306902 (PMC11379269; doi:10.1371/journal.pone.0306902)
Supplement: S2 File — (DOCX) [file pone.0306902.s002.docx]

|  | Female | | | |  | Male | | | |  |
| --- | --- | --- | --- | --- | --- | --- | --- | --- | --- | --- |
|  | death n = 159 | alive n = 1388 | OR (IC95%) | p |  | death n = 231 | alive n = 3104 | OR (IC95%) | p | p for interaction |
| Age ≥ 65 | 104 (65,4) | 554 (39,9) | 2,84 (1,99 – 4,09) | <0,001 |  | 127 (55,0) | 1066 (34,3) | 2,33 (1,77 – 3,09) | <0,001 | 0,374 |
| Emergency/urgency | 56 (35,2) | 53 (3,8) | 13,63 (8,71 – 21,38 | <0,001 |  | 72 (31,2) | 85 (2,7) | 16,04 (11,10 – 23,17) | <0,001 | 0,567 |
| Angina ≥ 4 | 72 (45,3) | 596 (42,9) | 1,09 (0,77 – 1,54) | 0,612 |  | 114 (49,4) | 1080 (34,8) | 1,82 (1,38 – 2,40) | <0,001 | 0,019* |
| NYHA 3-4 | 53 (33,3) | 220 (15,9) | 2,63 (1,80 – 3,82) | <0,001 |  | 77 (33,5) | 347 (11,2) | 3,98 (2,92 – 5,39) | <0,001 | 0,083 |
| AF | 4 (2,5) | 40 (2,9) | 0,86 (0,22 – 2,45) | >0,999 |  | 15 (6,5) | 87 (2,8) | 2,40 (1,26 – 4,28) | 0,004 | 0,092 |
| Previous PCI | 22 (13,8) | 225 (16,2) | 0,83 (0,49 – 1,34) | 0,493 |  | 34 (14,7) | 513 (16,5) | 0,87 (0,57 – 1,27) | 0,519 | 0,874 |
| PAD | 25 (15,7) | 109 (7,9) | 2,18 (1,30 – 3,54) | 0,002 |  | 51 (22,1) | 343 (11,1) | 2,27 (1,60 – 3,19) | <0,001 | 0,889 |
| Stroke | 14 (8,8) | 94 (6,8) | 1,32 (0,68 – 2,41) | 0,325 |  | 25 (10.8) | 225 (7,2) | 1,55 (0,96 – 2,41) | 0,051 | 0,677 |
| Diabetes | 77 (48,4) | 567 (40,9) | 1,35 (0,96 – 1,91) | 0,074 |  | 80 (34,6) | 994 (32,) | 1,12 (0,83 – 1,50) | 0,422 | 0,390 |
| Previous MI | 91 (57,2) | 629 (45,3) | 1,61 (1,14 – 2,28) | 0,005 |  | 111 (48,1) | 1533 (49,4) | 0,94 (0,71 – 1,24) | 0,733 | 0,014* |
| Recent MI | 46 (28,9) | 249 (17,9) | 1,86 (1,25 – 2,72) | 0,001 |  | 42 (18,2) | 567 (18,3) | 0,99 (0,68 – 1,41) | >0,999 | 0,015* |
| COPD | 40 (25,2) | 139 (10,0) | 3,01 (1,96 – 4,55) | <0,001 |  | 79 (34,2) | 471 (15,2) | 2,90 (2,14 – 3,90) | <0,001 | 0,877 |
| hipertension | 133 (83,6) | 1185 (85,4) | 0,87 (0,55 – 1,42) | 0,556 |  | 172 (74,5) | 2279 (73,4) | 1,05 (0,77 – 1,45) | 0,757 | 0,500 |
| Hemodialisis | 4 (2,5) | 14 (1,0) | 2,53 (0,59 – 8,19) | 0,104 |  | 11 (4,8) | 40 (1,3) | 3,82 (1,74 – 7,73) | <0,001 | 0,537 |
| CKD | 46 (28,9) | 100 (7,2) | 5,23 (3,42 – 7,92) | <0,001 |  | 73 (31,6) | 461 (14,9) | 2,64 (1,94 – 3,58) | <0,001 | 0,006* |
| Obesity | 38 (23,9) | 311 (22,4) | 1,08 (0,71 – 1,61) | 0,688 |  | 30 (13) | 352 (11,3) | 1,16 (0,75 – 1,75) | 0,453 | 0,803 |
| Smoking | 38 (23,9) | 379 (27,3) | 0,83 (0,55 – 1,23) | 0,396 |  | 60 (26,0) | 1084 (34,9) | 0,65 (0,47 – 0,89) | 0,006 | 0,324 |
| Dislipidemia | 48 (30,2) | 408 (29,4) | 1,03 (0,71 – 1,50) | 0,854 |  | 47 (20,3) | 845 (27,2) | 0,68 ( 0,48 – 0,95) | 0,025 | 0,091 |
| Anti-platelet | 70 (44,0) | 645 (46,5) | 0,90 (0,64 – 1,27) | 0,614 |  | 101 (43,7) | 1457 (46,9) | 0,87 (0,66 – 1,15) | 0,374 | 0,886 |
| B-bloq | 98 (61,6) | 1028 (74,1) | 0,56 (0,39 – 0,80) | 0,001 |  | 145 (62,8) | 2226 (71,7) | 0,66 (0,49 – 0,88) | 0,005 | 0,456 |
| Diuretic | 59 (37,1) | 355 (25,6) | 1,71 (1,19 – 2,44) | 0,002 |  | 67 (29) | 586 (18,9) | 1,75 (1,28 – 2,38) | <0,001 | 0,923 |
| Statin | 93 (58,5) | 918 (66,1) | 0,72 (0,51 – 1,02) | 0,060 |  | 150 (64,9) | 2149 (69,2) | 0,82 (0,61 – 1,10) | 0,184 | 0,554 |
| ACE inhibitor | 84 (52,8) | 642 (46,3) | 1,30 (0,92 – 1,83) | 0,130 |  | 111 (48,1) | 1372 (44,2) | 1,16 (0,88 – 1,53) | 0,272 | 0,616 |
| IABP | 35 (22,0) | 106 (7,6) | 3,40 (2,16- 5,29) | <0,001 |  | 51 (22,1) | 303 (9,8) | 2,61 (1,83 – 3,68) | <0,001 | 0,335 |
| LMD | 53 (33,3) | 363 (26,2) | 1,41 (0,97 – 2,02) | 0,058 |  | 84 (36,4) | 914 (29,4) | 1,36 (1,02 – 1,82) | 0,030 | 0,893 |
| FE < 40% | 42 (19,6) | 172 (80,4) | 2,54 (1,72 – 3,74) | <0,001 |  | 72 (13,1) | 479 (86,9) | 2,48 (1,85 – 3,33) | <0,001 | 0,928 |

|  |
| --- |
